# A Rare Presentation of Imperforate Hymen: A Case Report

**DOI:** 10.1155/2013/731019

**Published:** 2013-09-17

**Authors:** Beena Salhan, Olufunmilayo Theresa Omisore, Priyadarshi Kumar, John Potter

**Affiliations:** Northampton General Hospital, Cliftonville, Northampton NN1 5BD, UK

## Abstract

*Introduction*. Acute urinary retention in a child is rare. Haematocolpos can cause a mechanical obstruction, resulting in acute urinary retention. *Case Report*. A 12-year-old girl presented to the surgical department with a one-day history of acute urinary retention and suprapubic tenderness. She had not started menses but had described period-like pains every month for the past six months. On examination, she had a palpable bladder with over 500 mls of residual urine and a bluish-grey bulge posterior to her urethral meatus. An US scan showed a large mass posterior to her bladder resembling a haematocolpos, and this was confirmed with an MRI scan. She was catheterised and eventually underwent a hymenectomy using a cruciate incision. She made a good recovery postoperatively. *Conclusion*. In the case of a peripubertal female presenting with acute urinary retention, haematocolpos should be considered as a diagnosis.

## 1. Introduction

This is a case report of acute urinary retention as a result of an imperforate hymen causing haematocolpos. The incidence of imperforate hymen is 1 in 2000 girls, and approximately half of these will present with urinary retention [1]. Haematocolpos is a rare condition, where the vagina is filled with menstrual blood, caused by uterovaginal pathologies such as an imperforate hymen [2]. Most cases of imperforate hymen are sporadic in nature; however there have been reports of familial cases, where both recessive inheritance and dominant inheritance have been shown [3]. 

## 2. Case

In September 2012, a 12-year-old girl presented to the accident and emergency department with a one-day history of acute urinary retention associated with suprapubic pain and dysuria. There was no history of vomiting or a change in bowel habit. She reported cyclical abdominal cramping pains in the preceding six months but denied having started menses. Her birth history and developmental history were unremarkable. 

On examination, her abdomen was soft with mild tenderness suprapubically and in the left iliac fossa. Her bladder was palpable and she was noted to have a nontender bluish-grey bulge posterior to the urethra on examination of her external genitalia. Neurological examination was normal.

Urine dipstick was normal, and a urinary pregnancy test was negative. A bladder scan revealed over 500 mls of residual urine; therefore, a 10 Ch urinary catheter was inserted, which relieved her suprapubic pain. On repeat examination, the bladder was no longer palpable and a PR examination was normal with no palpable masses. Initial blood tests showed a mildly raised WCC at 11.7 and raised neutrophils at 10.15; all other blood results were unremarkable.

**Figure 1:**
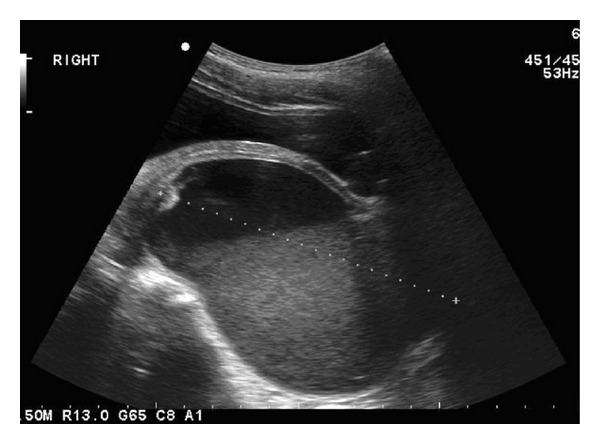
Ultrasound scan showing fluid-filled uterus.

An ultrasound scan of the kidneys showed an 11 × 7.8 × 8 cm fluid-filled mass lying posterior to the bladder, inseparable from and lying immediately inferior to the uterus (Figure 1). The mass had a fluid level, and findings were consistent with a hydrometra. The right kidney showed mild hydronephrosis. No other abnormal findings were detected.

**Figure 2:**
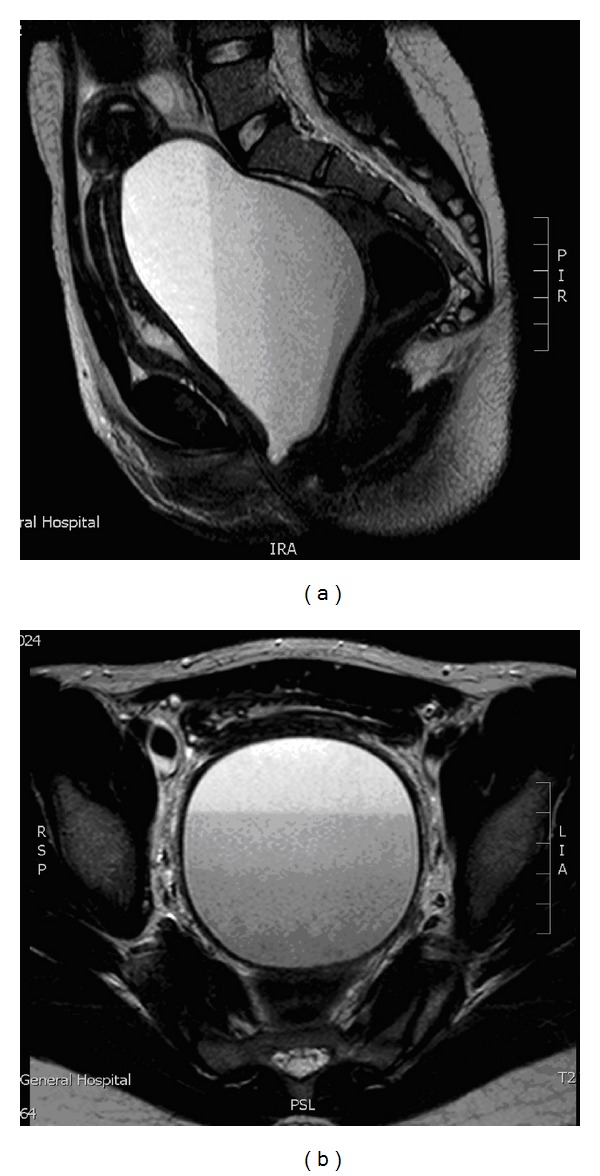
MRI scans showing the differential layers of fluid indicative of blood products.

Following the ultrasound findings, she was referred to the gynaecology department and underwent an MRI scan. This showed an 11 × 7.8 × 8 cm mass lying within the midline of the pelvis, which had several fluid layers indicating that it consisted of blood products (Figures 2(a) and 2(b)). Superiorly, the fluid was in continuation with a single uterine cavity, and inferiorly, it extended down to the perineum. Appearances were consistent with a hugely distended uterus filled with menstrual products.

Subsequently, she underwent a hymenotomy (using a cruciate incision) with drainage of her hydrocolpos. Postoperatively, she made a good recovery with a successful removal of the urinary catheter. Since returning home, she has started experiencing normal menses and has had no further urinary problems.

## 3. Discussion

Acute urinary retention is not a common presentation in children and is more common in males [4]. When young females present, the causes can include mechanical obstructions (urinary tract stones, urethral strictures, trauma to external genitalia, and imperforate hymen), neurological disorders, and urinary tract infection [4].

Imperforate hymen is a rare genital tract anomaly which has an incidence of about 1 in 2000 [1]. Acute urinary retention can subsequently occur due to the pressure effect imposed on the bladder and urethra [5]. 

This case serves to illustrate that in peripubertal females with amenorrhoea and acute urinary retention, even though uncommon, a diagnosis of haematocolpos should be considered and excluded.
